# A Comparative Analysis of Radiological Imaging and Surgical Treatments for Maxillary Artery Pseudoaneurysms, Based on a Literature Review and Our Clinical Experience

**DOI:** 10.3390/biomedicines13061410

**Published:** 2025-06-09

**Authors:** Kinga Samól, Adam Michcik, Barbara Wojciechowska, Adam Polcyn, Łukasz Garbacewicz, Barbara Drogoszewska

**Affiliations:** 1 Department of Maxillofacial Surgery, University Clinical Centre in Gdańsk, Mariana Smoluchowskiego 17, 80-214 Gdansk, Poland; ksamol@uck.gda.pl (K.S.); barbara.wojciechowska@gumed.edu.pl (B.W.); adampolcyn@gumed.edu.pl (A.P.); lgarbacewicz@gumed.edu.pl (Ł.G.); drog@gumed.edu.pl (B.D.); 2 Department of Maxillofacial Surgery, Faculty of Medicine, Medical University of Gdansk, Mariana Smoluchowskiego 17, 80-214 Gdansk, Poland

**Keywords:** pseudoaneurysms, maxillary artery, endovascular embolization, surgical resection, hemorrhage, angiography

## Abstract

**Background/Objectives**: A pseudoaneurysm forms as a result of disruption of all artery wall layers. In the head and neck, they are most commonly found in the maxillary artery. Due to their location and associated symptoms, detailed radiological imaging is necessary to determine the nature and extent of lesions. Various treatment methods are available. **Methods**: To systematize symptoms, diagnostics, and treatment methods, a literature review from databases spanning 2014 to 2024 was conducted, with 30 articles included in the study. **Results**: The factors that caused MAPs included facial trauma (n = 33; 66%), iatrogenic surgical procedures (n = 14; 28%), head and neck radiotherapy (n = 1; 2%), infection (n = 1; 2%), and one case due to an idiopathic factor (n = 1; 2%). Diagnostic imaging included computed tomography with contrast, magnetic resonance imaging, and angiography. Treatment methods used: endovascular embolization (n = 44; 88%), surgical resection (n = 3; 6%), cauterization (n = 2; 4%), and compression tamponade (n = 1; 2%). Interestingly, three of the cases were treated with endoscopic access (6%). **Conclusions**: It can be concluded that the most common cause of MAPs is trauma to the facial skeleton, and the most frequently used treatment method is endovascular embolization. Given the need for detailed MAP imaging and treatment in specialized invasive radiology departments, patients with MAPs should be treated in multidisciplinary clinical centers.

## 1. Introduction

An aneurysm is an irreversible dilatation of an artery [1]. True aneurysms include all layers of the arterial walls (i.e., tunica externa, media, and intima), whereas pseudoaneurysms may have one or two components [1,2,3,4]. An aneurysm may be caused by injuries that rupture the full thickness of the arterial wall, leading to extravasation of blood into surrounding tissue, producing a pulsatile hematoma [1,5]. A clot forms and begins to organize. During the process of repairing a damaged vessel, the cellular components form an aneurysmal sac, and the inflammation around the hematoma forms a fibrous capsule [4]. The center of the clot becomes liquid. The arterial pressure or “jetting” of blood through the sac causes the lesion to pulsate and a murmur is audible on auscultation [4]. Pseudoaneurysms usually appear 1 to 8 weeks after vessel injury [3].

The most typical locations of pseudoaneurysms are the three branches of the external carotid system, namely the superficial temporal, facial, and maxillary arteries [4,6].

The maxillary artery (MA) is a terminal branch of the external carotid artery (ECA). A sizeable area of the head and neck, including surrounding soft tissues, the oral and sinonasal cavities, the dura mater, and various cranial nerves, is supplied by this [7].

According to its classical description, the MA begins behind the neck of the mandible, in the parotid gland, and runs upwards and forwards, through the infratemporal region parallel to and below the auriculotemporal nerve, then reaches the lateral pterygoid muscle, running along its lower edge. Then it reaches the infratemporal fossa, where it turns and enters the pterygopalatine fossa through the pterygomaxillary fissure. Subsequently it runs under the maxillary nerve and in front of the pterygopalatine ganglion, finally entering through the sphenopalatine foramen, where it ends as the sphenopalatine artery [8].

The MA typically gives off 14 collateral branches and one terminal branch (pterygopalatine artery) and is divided into three parts: first, the mandibular part; second, the pterygoid part; and third, the pterygopalatine part [8,9]. In the second section, it can run laterally/superficially or medially/deeply to the lateral pterygoid muscle.

According to some studies, the MA may pass through the inferior head of the lateral pterygoid muscle and should then be classified as “intermediate type” [8]. Maeda et al. [10] noted that this variant is quite rare. According to Arimoto et al. [11], in most cases (97%) the MA runs laterally/superficially to the lateral pterygoid muscle, and only in 3% of cases is its route deep [8]. Morton and Khan classified four types of branching patterns of the MA in the pterygopalatine fossa (the third segment of the MA) depending on the forking model of the sphenopalatine artery (SPA) and the descending palatine artery (DPA) [12]. In that study, 100 hemispheres were categorized into the four types of bifurcation pattern based on the degree of angulation between the DPA and SPA arteries at the splitting point: a Y-shaped pattern (bifurcation angle close to 180 degrees), a T-shaped pattern (bifurcation angle greater than 90 degrees), an intermediate pattern (bifurcation angle close to 90 degrees), and an M pattern (bifurcation with angulation close to 0 degrees) [8,12].

A pseudoaneurysm (PA) of the ECA branches is relatively rare, because in most locations the ECA ramifications are protected from injury by an adequate soft tissue buffer. In addition, trauma of these anatomical structures usually results in total transection rather than partial laceration of the blood vessel [13]. According to this, the lateral type of maxillary artery, which follows the most superficial route to the lateral pterygoid muscle, appears to be predisposed to pseudoaneurysm development.

Most aneurysms of the maxillary artery are present in its terminal segments, whereas they are rare in its mandibular part [1]. Pseudoaneurysms in the external carotid artery (ECA) branches are commonly secondary to facial trauma and fractures, leading to vascular injury [2]. Other etiologies, albeit less frequent, include needle aspiration procedures, infections/abscesses, arteriosclerosis, cystic medial necrosis, fibromuscular dysplasia, congenital anomalies, radiation vasculitis, radiotherapy, poor nutrition, and malignancy [2,6,14]. In the literature, pseudoaneurysms of the branches of the external carotid artery have been also reported as a result of iatrogenic injuries during oral/facial surgeries, such as tonsillectomy, neck dissection, open reduction and internal fixation (ORIF) of a mandibular fracture, Le Fort I osteotomies, temporomandibular joint surgeries, and mandibular vertical ramus osteotomies [14]. The clinical symptoms of PAs may be varied, including a pulsatile mass, craniocervical pain, bleeding, dysphagia, hoarseness, and neurologic deficits [6]. Nicholson et al. [15] reported a case of central retinal artery occlusion in a 44-year-old male caused by emboli from a non-traumatic maxillary artery pseudoaneurysm.

Suspicion of a posttraumatic PA should be raised in every case of a pulsating swelling with an audible systolic bruit observed during clinical examination, resulting from a previous injury [1]. PA treatment includes various options such as surgical excision or endovascular embolization [16]. The proper choice usually depends on the size and location of the lesion, as well as the patient’s intraoperative mortality risk and health status [16,17]. In percutaneous embolization, metallic coils, polyvinyl alcohol particles, and absorbable gelatin sponges are used to occlude. In case of failure of these methods, surgical exploration and excision are undertaken [17].

This study evaluated radiological imaging methods and the management of patients with MAPs and assessed whether treatment methods depend on the etiology of MAPs.

## 2. Materials and Methods

**Figure 1:**
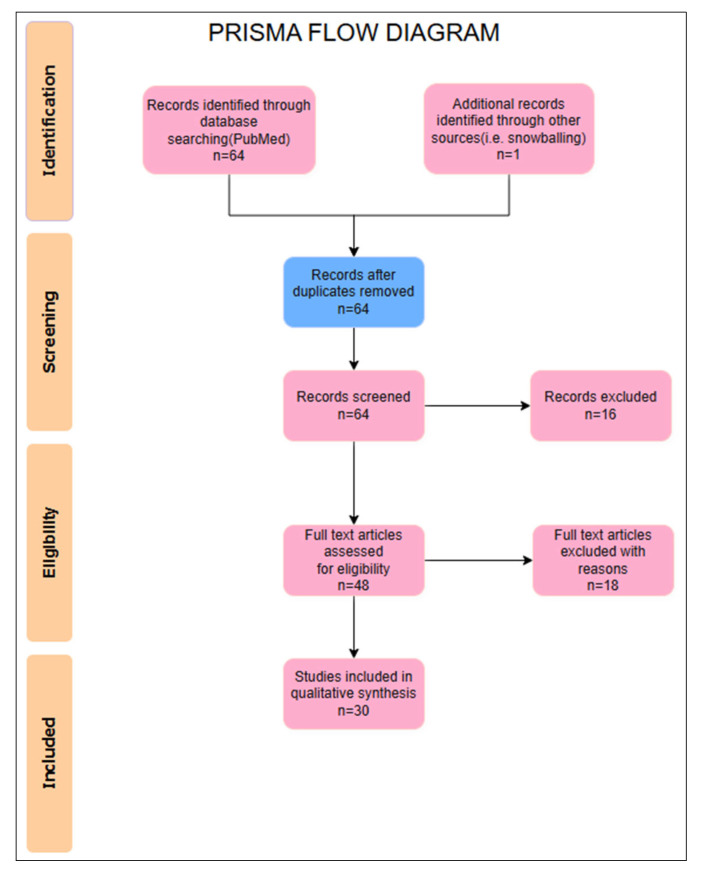
Prisma flow diagram.

A literature review was performed to assess the current state of knowledge regarding the etiology and treatment methods of maxillary artery pseudoaneurysms. The search was conducted on data obtained from a literature review of the most popular database, PubMed (Medline). We conducted an electronic search of the PubMed database between 1 July 2014 and 1 July 2024. The following keywords were used during the search process: “internal maxillary artery”, ’’pseudoaneurysm”, “management”, “surgical techniques”, and “endovascular embolization”. These keywords were combined using Boolean operators (AND, OR) to ensure a comprehensive search that included all pertinent studies. Only articles describing clinical cases or case series that included details on surgical treatment techniques and etiological factors were included. Exclusion criteria included papers that were not published in English or were not available in full text. Two researchers assessed the quality of the studies. The flow diagram is presented in Figure 1.

The identified publications were analyzed for the etiology of pseudoaneurysms and the surgical techniques used. The results were compiled into a table and analyzed quantitatively to determine the most commonly used treatment methods. It was also assessed whether radiological imaging and treatment methods depend on the etiology of MAPs. Additionally, two cases from our clinical experience were included and analyzed according to the same criteria. After analyzing 64 articles available in the PubMed database, we decided to include 30 of them in our comparative analysis.

## 3. Results

Table 1 summarizes the patients’ baseline characteristics, including age, gender, MAP etiology, current symptoms, and the time that had passed between the etiological factor occurring and the first symptoms appearing.

The analysis included 50 cases of pseudoaneurysms occurring within the maxillary artery, sourced from the PubMed publication database for 2014–2024. Data on the causes and treatment methods are compiled in Table 2.

According to the data presented above, the main etiological factor causing maxillary artery pseudoaneurysms was facial trauma (n = 33; 66%). Fourteen out of fifty cases were caused by iatrogenic surgical procedures (28%).

Other etiological factors included consequences of head and neck radiotherapy (n = 1; 2%), complications following infection (n = 1; 2%), and an idiopathic factor (n = 1; 2%). The most common treatment technique used in the presented cases was endovascular embolization (n = 44; 88%). Surgical resection was performed in three cases (6%), two with cauterization (4%) and one with compression tamponade (2%). Of the fifty described cases, endoscope treatment was used in three cases (6%).

**Figure 2:**
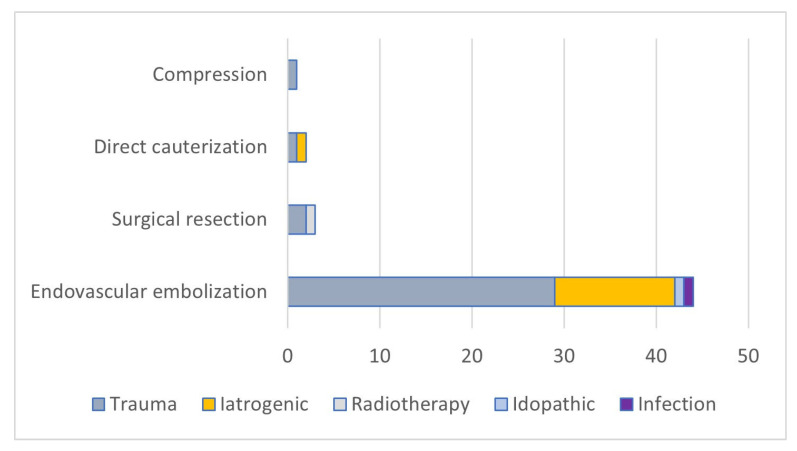
Relationship between the cause of the injury and the type of treatment.

**Figure 3:**
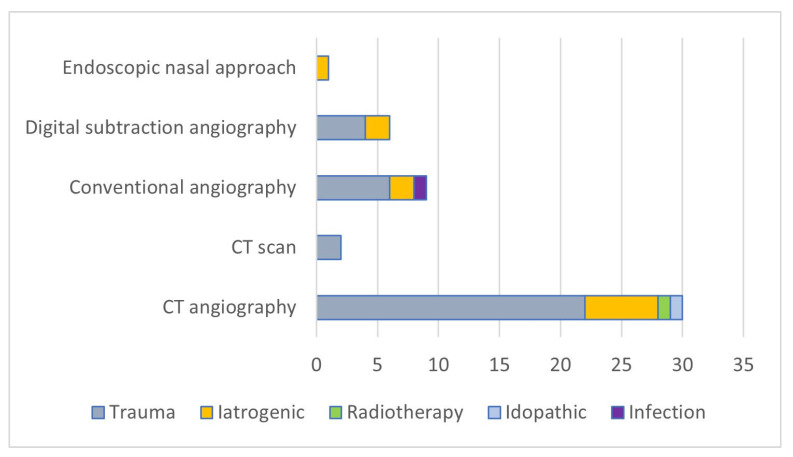
Relationship between the cause of the injury and the type of radiological image method.

We obtained the results presented in Figure 2 and Figure 3 by assessing the radiological imaging method used and the treatment depending on the etiology of the MAP formation.

### Case Series

**Figure 4:**
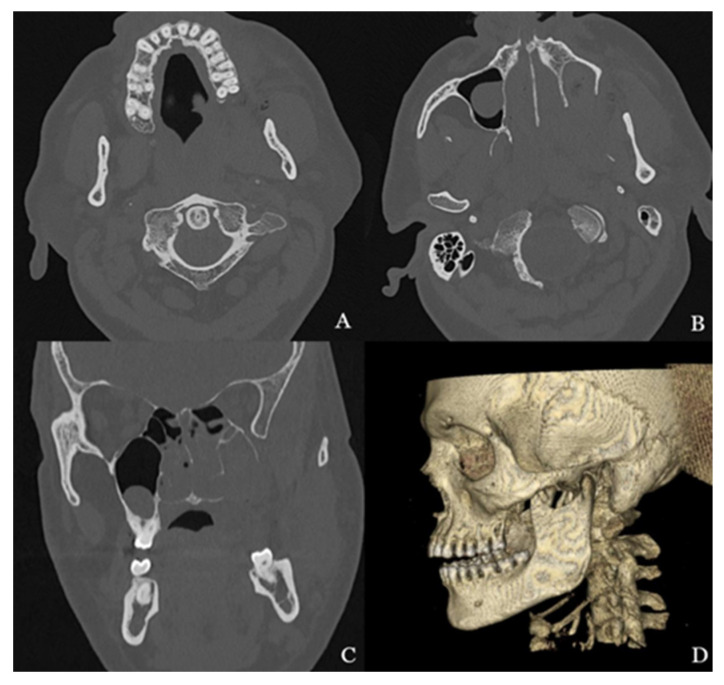
CT scans showing the bone defect in the maxilla after the extraction of tooth 28 ((**A**,**B**) axial sections, (**C**) coronal section), and a 3D reconstruction of the defect (**D**). The vascular abnormalities are not visible.

A 51-year-old male, with no significant systemic medical history, presented to the Clinical Emergency Department due to bleeding following the odontectomy of tooth 28. The procedure had been performed two weeks earlier. The patient reported that the tooth had been removed along with a fragment of bone, and an oral–antral communication detected after the procedure had been repaired. A CT scan, performed without and with contrast, revealed defects in the inferior, medial, and lateral walls of the left maxillary sinus, as well as the palatal plate. Fresh blood was detected in the sinus. Details can be seen in Figure 4.

**Figure 5:**
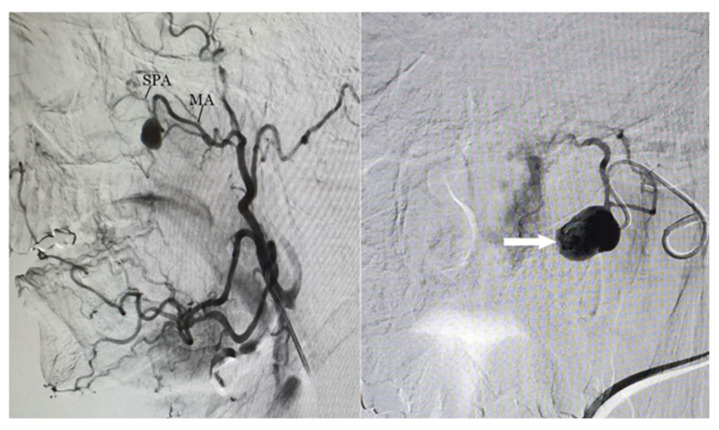
Angiogram showing a pseudoaneurysm (indicated by an arrow) at the origin of the sphenopalatine artery from the maxillary artery; SPA—sphenopalatine artery, MA—maxillary artery.

The overall findings were consistent with active bleeding from the left sphenopalatine artery. Due to the worsening condition of the patient after the transfusion of two units of packed red blood cells (PRBCs), the patient was qualified for emergency embolization. Under general anesthesia and ultrasound guidance, a vascular catheter was introduced into the right femoral artery, reaching the left external carotid artery. Angiography revealed the presence of a pseudoaneurysm approximately 2 cm in size at the origin of the sphenopalatine artery from the maxillary artery, as shown in Figure 5.

**Figure 6:**
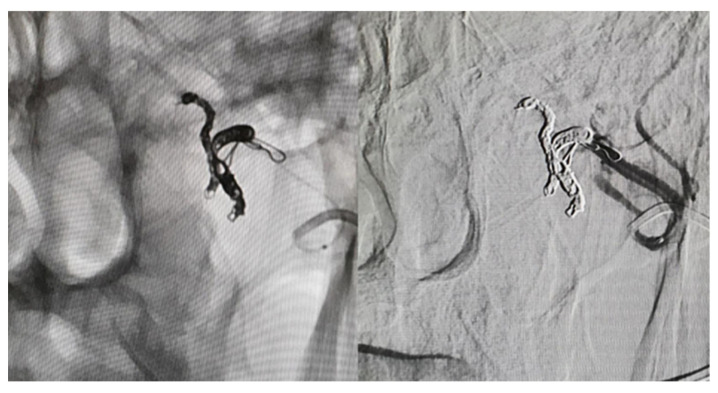
Post-trapping state of the pseudoaneurysm. Embolization of segments of the sphenopalatine artery is visible, using platinum coils (two 2 × 8 mm coils, one 2 × 6 mm coil, and two 2.5 × 8 mm coils), with no blood flow in the aneurysm. Embolization of the maxillary artery segment near the pseudoaneurysm was carried out using an 8 × 30 mm coil.

In the first stage of the procedure, embolization of the proximal segment of the sphenopalatine artery was performed using platinum coils (two 2 × 8 mm coils, one 2 × 6 mm coil, and two 2.5 × 8 mm coils). Subsequently, embolization of the maxillary artery segment near the pseudoaneurysm was carried out using an 8 × 30 mm coil. Control angiography confirmed effective trapping of the pseudoaneurysm sac, as shown in Figure 6.

On the second day following the embolization procedure, the patient, in good general and local condition, was discharged home with appropriate recommendations. During a two-month follow-up period, the patient did not report any recurrent episodes of bleeding. The treatment was successfully completed.

**Figure 7:**
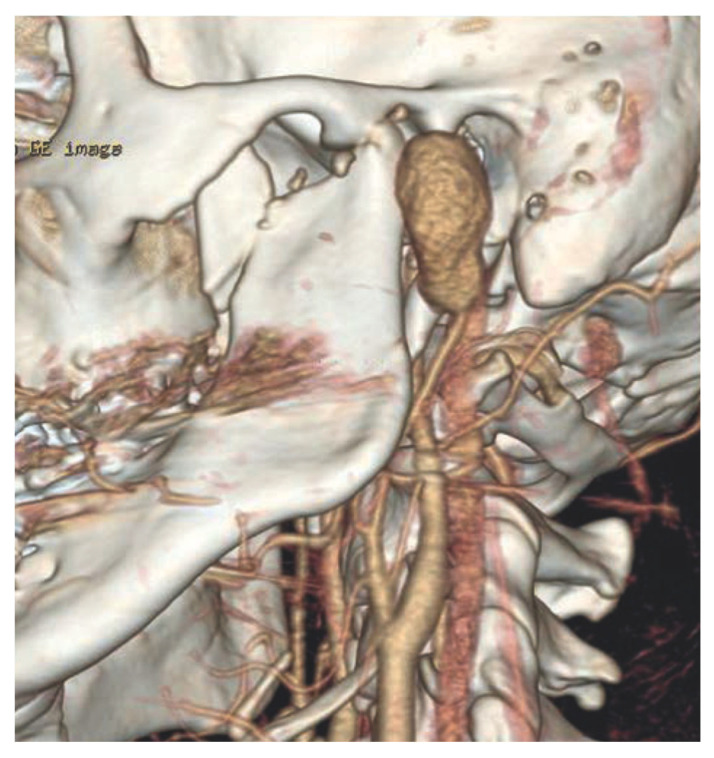
CT angiography. Visible pseudoaneurysm and fracture of the condylar head and coronoid process.

The second case observed in our department involved a 20-year-old male patient who was admitted with progressive swelling and facial asymmetry in the left preauricular region. The patient had no significant medical history. One month prior, he had sustained bilateral condylar process fractures as a result of a fall from height. The fractures were managed conservatively using orthopedic methods, including intermaxillary fixation and dental splints. Approximately one month after the initial hospitalization, the patient presented to the Oral Surgery Outpatient Clinic with a gradually enlarging swelling in the left preauricular area, initially suspected to be an abscess. Incision of the overlying skin resulted in massive hemorrhage, which was successfully controlled by surgical suturing of the wound edges. The patient was subsequently referred to the Department of Maxillofacial Surgery. Physical examination revealed facial asymmetry, increased tension in the left preauricular region, and a palpable pulsation. No other abnormalities were noted in the clinical evaluation. Ultrasonographic imaging, followed by computed tomography angiography (CTA), demonstrated a pseudoaneurysm measuring 31 × 20 × 21 mm, located at the level of the origin of the maxillary artery from the left external carotid artery, as shown in Figure 7.

**Figure 8:**
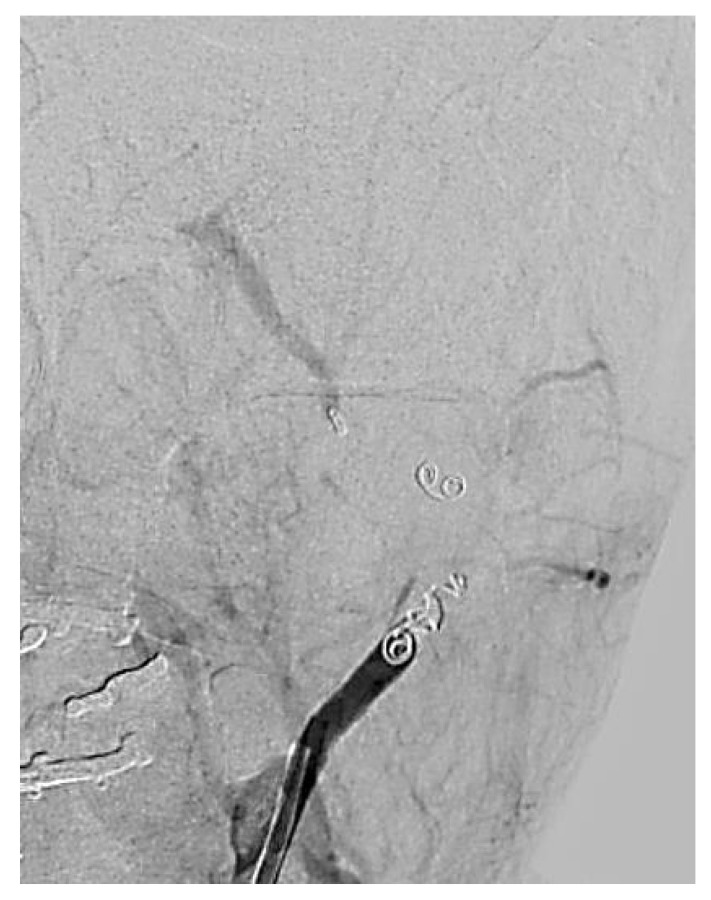
An angiogram showing the lack of blood flow in a pseudoaneurysm.

Interventional treatment was carried out by accessing the femoral artery and advancing a microcatheter through the external carotid artery to the maxillary artery. Endovascular embolization was performed using a titanium coil, successfully occluding the distal segment of the maxillary artery. Control arteriography confirmed complete vessel occlusion, as shown in Figure 8.

The patient was discharged in good general and local condition on the second postoperative day. At a one-month follow-up, the patient was asymptomatic, with complete resolution of clinical signs and regression of the pseudoaneurysm.

## 4. Discussion

### 4.1. Radiological Diagnosis

There are several imaging methods that can confirm diagnosis, e.g., a computed tomography (CT) scan with contrast. This imaging modality is recommended because it allows us to visualize anatomical details of vascular injuries with high accuracy [42]. Although contrast-enhanced CT provides the dimensions of the lesion and its relation to surrounding structures, it may not clearly demonstrate the vascular abnormalities in partially developed pseudoaneurysms [14]. Doppler ultrasound imaging is the most commonly used non-invasive test and a helpful tool in the evaluation of a PA, revealing a fusiform or saccular eccentric dilatation of the affected artery [43]. The ability to detect turbulent flow and vasodilation with 95% accuracy in PAs is the main advantage of this procedure [44]. However, in relation to lesions located deeper, the effectiveness of Doppler ultrasound decreases [13,15].

Magnetic resonance imaging (MRI) or conventional angiography may still be necessary. The characteristics of ECA PAs on MRI depend on the presence of an arteriovenous fistula (AVF). In patients without an AVF, the MAP presented with isointense signal intensity on the T1-weighted image (WI), hyperintense signal intensity on the T2WI, and significant enhancement on the T1WI after contrast agent administration [43].

Angiography techniques as a non-routine diagnostic method for MAPs should be considered after the use of non-invasive imaging techniques, such as duplex ultrasound imaging or MRI, after evaluation of the lesion and when endovascular treatment is a viable option [43].

Our Department of Maxillofacial Surgery encountered two cases of maxillary artery pseudoaneurysms. The first case, documented in 2019, involved the utilization of ultrasonography and angiography for diagnostic evaluation [45]. In contrast, the second case, in 2024, relied on computed tomography (CT) scanning for diagnosis. Both methodologies proved effective in accurately delineating the location and dimensions of the pseudoaneurysms.

Despite the limitations of CT scanning, we continue to use it as the initial screening test, complementing it with MRI or conventional angiography depending on the clinical situation [43,46,47].

### 4.2. Differential Diagnosis

The differential diagnosis encompasses a variety of conditions, including hematoma, abscess, inflamed lymph node, lipoma, cyst, and pleomorphic adenoma. Each presents distinct clinical and imaging characteristics that must be carefully evaluated to arrive at an accurate diagnosis [14,43]. In the case of surgical treatment, the diagnosis of a pseudoaneurysm is established by the pathologist, who possesses the expertise to microscopically differentiate between true and false aneurysms based on the involvement of the various layers of the vessel wall [14]. However, in most cases, the radiological image in conjunction with clinical symptoms is pathognomonic.

### 4.3. Treatment Methods

MAP treatment encompasses a range of surgical and endovascular options. Surgical resection is not always feasible due to the limited access to deep located lesions [20,48]. In addition, surgery increases the risk of nerve damage and may cause cosmetic defects such as facial scars [18]. Catheter embolization is a safe, quick, and effective technique that allows us to avoid the morbidity associated with extensive surgery. Endovascular embolization involves either the use of occlusive material or the placement of a stent (covered or not) across the PA base [13,18]. Various agents have been used for this kind of therapy, e.g., metallic coils, polyvinyl alcohol particles, *n*-butyl cyanoacrylate (NBCA), polymers (Onyx, SQUID), and absorbable sponge gels [18,43].

Metallic coils are permanent embolic agents, with or without attached fibers. Coils are positioned in the vessel lumen proximal to the PA, which arrests the flow. Fibers attached to the coils increase the thrombotic effect. NBCA, Onyx, and SQUID penetrate deeper into the vessel and may enter the venous system [18]. The use of metallic coils to occlude the parent artery is considered an effective procedure. A disadvantage of this method is the risk of recurrent bleeding resulting from retrograde filling of the PA via indirect collateral circulation. The filling of the MAP sac with coils may cause rupture of the aneurysm wall, leading to coil migration beyond the target lesion. Acute complications of endovascular treatment include distal thromboembolic events (occlusion of the central retinal artery, ischemic stroke due to potential anastomosis between the MA and the ophthalmic artery) and local tissue damage. Furthermore, perforations, glued vein, microcatheter fracture, and vessel dissection or branch occlusion were reported in the literature [13,17,18]. The analysis of surgical techniques used in treating maxillary artery pseudoaneurysms highlights the growing role of endovascular embolization as the primary therapeutic approach. Our findings indicate that embolization was the most frequently performed procedure (86.96% of cases), consistent with the current literature, which underlines its effectiveness in achieving hemostasis while minimizing surgical trauma. Although still used in selected cases, traditional open surgical techniques appear to be reserved for situations where embolization is not possible or has failed [16,41]. The treatment method choice depends on multiple factors, including the size and location of the pseudoaneurysm, the patient’s clinical condition, and the availability of endovascular expertise [16]. Embolization has gained popularity due to its minimally invasive nature, shorter recovery time, and lower risk of complications than open surgery. However, potential risks such as non-target embolization, rupture of the arterial vessel, in particular the pseudoaneurysm wall, stroke caused by embolic material, necrosis of the maxillary region, peripheral facial nerve paralyses, tooth loss, and other minor temporary complications should be taken into account [16]. Our case study further supports the literature by demonstrating successful hemostasis following embolization, reinforcing its role as the first-line treatment. Nevertheless, rare cases may require a combined approach, integrating endovascular and surgical techniques, particularly in complex or recurrent pseudoaneurysms [49]. One limitation of this study is the heterogeneity of the available literature, as case reports and case series often lack standardized outcome measures. Additionally, long-term follow-up data are limited, making it challenging to evaluate the durability of various treatment approaches. Further research, including prospective studies and multicenter analyses, is necessary to optimize management strategies and improve patient outcomes. Given the need for a multidisciplinary approach, patients with maxillary artery pseudoaneurysms should be treated in high-reference centers equipped with both endovascular and surgical expertise. Future studies should focus on refining embolization techniques, identifying optimal embolic agents, and evaluating long-term efficacy to establish standardized treatment guidelines.

## 5. Conclusions

According to the literature review above, facial trauma is the most common cause of maxillary artery pseudoaneurysms, followed by surgical procedures. A CT scan is the most frequently used diagnostic technique, enabling precise localization of the pseudoaneurysm and its anatomical relationship with surrounding structures. Treatment depends on many factors. However, in most of the described cases, embolization therapy was performed, indicating the need to treat patients diagnosed with maxillary artery pseudoaneurysms at high-reference centers with multidisciplinary teams of experienced physicians.

## Figures and Tables

**Table 1:** Characteristics of the study group.

Characteristic		N	%
Gender	Male	38	76
	Female	12	24
Age	10s	4	8
	20s	14	28
	30s	11	22
	40s	9	18
	50s	6	12
	60s	4	8
	70s	2	4
Etiology of the MAPs	Trauma	33	66
	Radiotherapy	1	2
	Iatrogenic	14	28
	Idiopathic	1	2
	Infection	1	2
Symptoms	Swelling	27	54
	Bleeding	27	54
	Trismus	1	2
	Facial palsy	2	4
	Dental maloclusion	1	2
	Nasal obstruction	2	4
	Pain	10	20
Latency time	Unknown	17	34
	Few hours	3	6
	1–10 days	12	24
	11–30 days	8	16
	1–12 months	8	16
	1–10 years	1	2
	>10 years	1	2

**Table 2:** Summary of described maxillary artery pseudoaneurysm cases with etiology and treatment methods.

Nr.	Author	Year	Etiology	Treatment	Radiological Imaging Method
1.	Katakol et al. [4]	2014	Trauma (fracture of the mandibular condylar process)	Intravascular embolization	CT angiography
2.	Kamath et al. [17]	2014	Head and neck radiotherapy	Surgical resection	CT angiography
3.	Wang et al. [13]	2015	Trauma (13 of 17 cases) Iatrogenic (3 of 17 cases) Idiopathic (1 of 17 cases)	Intravascular embolization	CT angiography
4.	Chakrabarty et al. [1]	2015	Trauma (fracture of the mandibular condylar process)	Surgical resection	CT angiography
5.	Soh et al. [18]	2015	Trauma (fracture of the mandibular condylar process)	Intravascular embolization	CT scan
6.	Thakkur et al. [19]	2015	Trauma (fracture of the mandibular condylar process)	Surgical resection	CT angiography
7.	Alonso et al. [5]	2016	Trauma (gunshot)	Intravascular embolization	Conventional angiography
8.	Nastro Siniscalchi et al. [20]	2016	Trauma (Le Fort III fracture)	Intravascular embolization	CT angiography and conventional angiography
9.	Lee et al. [21]	2016	Iatrogenic (endoscopic sinus procedure)	Intravascular embolization	CT angiography
10.	Gold et al. [22]	2016	Trauma (gunshot)	Intravascular embolization	CT angiography
11.	Debelmas et al. [23]	2017	Trauma (Le Fort I fracture)	Intravascular embolization	CT angiography
12.	Shan JP et al. [24]	2017	Iatrogenic (endoscopic sinus procedure)	Intravascular embolization	Digital subtraction angiography
13.	Tao et al. [25]	2017	Trauma (zygomatico-maxillo-orbital fracture)	Intravascular embolization	CT angiography and digital subtraction angiography
14.	Moro et al. [6]	2018	Trauma (fracture of the mandibular condylar process)	Intravascular embolization	Conventional angiography
15.	Neres et al. [26]	2018	Trauma (gunshot)	Intravascular embolization	Conventional angiography
16.	Kim et al. [27]	2019	Iatrogenic (injection in the masseter muscle)	Intravascular embolization	Conventional angiography
17.	Al-Saadi et al. [28]	2019	Trauma (3 cases)	Intravascular embolization	Digital subtraction angiography
18.	Chaudhary et al. [29]	2019	Infection (complication of malignant otitis externa)	Intravascular embolization	Conventional angiography
19.	Chun et al. [30]	2019	Trauma (zygomatico-maxillo-orbital fracture) (2 cases)	Intravascular embolization	Conventional angiography
20.	Rawat et al. [31]	2019	Iatrogenic (surgical extraction of impacted upper third molar)	Intravascular embolization	Digital subtraction angiography
21.	Maleux et al. [32]	2019	Iatrogenic (SARPE procedure)	Intravascular embolization	CT angiography
22.	Park et al. [33]	2019	Iatrogenic (Le Fort I osteotomy)	Direct cauterization with endoscopic transnasal access	Endoscopic nasal approach
23.	ManojKumar et al. [34]	2021	Iatrogenic (Le Fort I osteotomy)	Intravascular embolization	Conventional angiography
24.	Kumar et al. [35]	2021	Iatrogenic (Le Fort I osteotomy with simultaneous BSSO procedure)	Intravascular embolization	CT angiography
25.	Słotwińska et al. [36]	2021	Trauma (bilateral fracture of the mandibular condylar process)	Intravascular embolization	CT angiography
26.	Hwang et al. [37]	2021	Trauma (zygomatico-maxillo-orbital fracture)	Compression tamponade using endoscopic access	CT scan
27.	Miller et al. [38]	2022	Trauma (zygomatico-maxillo-orbital fracture)	Direct cauterization with endoscopic transnasal access	CT angiography
28.	Lim et al. [39]	2022	Trauma (zygomatico-maxillo-orbital fracture)	Intravascular embolization	Conventional angiography
29.	Tokuyama et al. [40]	2023	Iatrogenic (surgical extraction of impacted upper third molar) (2 cases)	Intravascular embolization	CT angiography
30.	Botella-Casas et al. [41]	2024	Iatrogenic (sagittal osteotomy)	Intravascular embolization	CT angiography

## Data Availability

The raw data supporting the conclusions of this article will be made available by the authors on request.

## References

[1] Chakrabarty S., Majumdar S.K., Ghatak A., Bansal A. (2015). Management of Pseudoaneurysm of Internal Maxillary Artery Resulting from Trauma. J. Maxillofac. Oral Surg..

[2] Niazi M.H., El-Ghanem M., Al-Mufti F., Wajswol E., Dodson V., Abdulrazzaq A., Sami T., Nuoman R., Aziz S., Gandhi C.D. (2018). Endovascular Management of Epistaxis Secondary to Dissecting Pseudoaneurysm of the Descending Palatine Artery Following Orthognathic Surgery. J. Vasc. Interv. Neurol..

[3] Zachariades N., Rallis G., Papademetriou G., Papakosta V., Spanomichos G., Souelem M. (2001). Embolization for the Treatment of Pseudoaneurysm and Transection of Facial Vessels. Oral Surg. Oral Med. Oral Pathol. Oral Radiol. Endod..

[4] Katakol B., Govindaraj E. (2014). Pseudoaneurysm of the Internal Maxillary Artery Following Mandibular Condylar Fracture. Ann. Maxillofac. Surg..

[5] Alonso N., De Oliveira Bastos E., Massenburg B.B. (2016). Pseudoaneurysm of the Internal Maxillary Artery: A Case Report of Facial Trauma and Recurrent Bleeding. Int. J. Surg. Case Rep..

[6] Moro A., Todaro M., Pedicelli A., Alexandre A., Pelo S., Doneddu P., Gasparini G., Garagiola U., D’Amato G., Saponaro G. (2018). Pseudoaneurysm of the Internal Maxillary Artery Secondary to Subcondylar Fracture: Case Report and Literature Review. J. Surg. Case Rep..

[7] Akiyama O., Güngör A., Middlebrooks E.H., Kondo A., Arai H. (2018). Microsurgical Anatomy of the Maxillary Artery for Extracranial-Intracranial Bypass in the Pterygopalatine Segment of the Maxillary Artery. Clin. Anat..

[8] Ottone N.E., Sandoval C., Cid-Gutierrez P., Vásquez-Balboa M.L., Tubbs R.S., Fuentes R. (2021). Systematic Review and Meta-Analysis of the Anatomy of the Maxillary Artery Using the Anatomical Quality Assurance (AQUA) Checklist. Surg. Radiol. Anat..

[9] Hussain A., Binahmed A., Karim A., Sándor G.K.B. (2008). Relationship of the Maxillary Artery and Lateral Pterygoid Muscle in a Caucasian Sample. Oral Surg. Oral Med. Oral Pathol. Oral Radiol. Endodontology.

[10] Maeda S., Aizawa Y., Kumaki K., Kageyama I. (2012). Variations in the Course of the Maxillary Artery in Japanese Adults. Anat. Sci. Int..

[11] Arimoto S., Hasegawa T., Okamoto N., Shioyasono A., Tateishi C., Akashi M., Suzuki H., Furudoi S., Komori T. (2015). Determining the Location of the Internal Maxillary Artery on Ultrasonography and Unenhanced Magnetic Resonance Imaging before Orthognathic Surgery. Int. J. Oral Maxillofac. Surg..

[12] Morton A.L., Khan A. (1991). Internal maxillary artery variability in the pterygopalatine fossa. Otolaryngol. Head Neck Surg..

[13] Wang D., Su L., Han Y., Fan X. (2015). Embolization Treatment of Pseudoaneurysms Originating from the External Carotid Artery. J. Vasc. Surg..

[14] Shetty N.K., Shandilya R., Pawar S., Gadre P.K., Gadre K., Singh D. (2015). Management of Late Post-Traumatic Facial Artery Pseudoaneurysmal Cyst: Review of Literature. J. Maxillofac. Oral Surg..

[15] Nicholson A.T., Sullivan M.A., Corliss B.M. (2023). Maxillary Artery Pseudoaneurysm Causing Retinal Artery Occlusion. Am. J. Ophthalmol. Case Rep..

[16] Piccioni A., Vaccaro V., Manca F., Nonno C., Zanza C., Savioli G., Candelli M., Covino M., Franceschi F. (2022). Management of Maxillary Artery Pseudoaneurysm in Emergency Department: A Narrative Review. Clin. Ter..

[17] Kamath G., Naalla R., Pai V.B., Narayanan R. (2014). Left Maxillary Artery Pseudoaneurysm: A Rare and Late Postoperative Complication after Head and Neck Cancer Treatment. BMJ Case Rep..

[18] Soh H.Y., Muda A.S., Jabar N.A., Nordin R., Nabil S., Ramli R. (2015). Non-Pulsatile Traumatic Pseudoaneurysm of the Internal Maxillary Artery Following Trauma to Mandible. Oral Maxillofac. Surg..

[19] Thakkur R.K., Kapadia S., Merchant R. (2015). Traumatic Internal Maxillary Artery Pseudoaneurysm with a Malunited Mandibular Fracture. J. Maxillofac. Oral Surg..

[20] Nastro Siniscalchi E., Catalfamo L., Pitrone A., Papa R., Famà F., Lo Giudice G., Cervino G., Cicciu M., De Ponte F.S. (2016). Traumatic Pseudoaneurysm of the Internal Maxillary Artery: A Rare Life-Threatening Hemorrhage as a Complication of Maxillofacial Fractures. Case Rep. Med..

[21] Lee E.J., Hwang H.J., Kim K.S. (2016). Pseudoaneurysm in the Internal Maxillary Artery Occurring after Endoscopic Sinus Surgery. J. Craniofacial Surg..

[22] Gold M. (2016). Partially Thrombosed Internal Maxillary Pseudoaneurysm after Gunshot Wound. Craniomaxillofac. Trauma Reconstr..

[23] Debelmas A., Lanciaux S., Schouman T. (2017). A Case of Epistaxis. J. Stomatol. Oral Maxillofac. Surg..

[24] Shan J.P., Xu X.J., Wu J., Ji W.B. (2017). Transcatheter arterial embolization in the treatment of maxillary artery pseudoaneurysm hemorrhage: A case report. Lin Chuang Er Bi Yan Hou Tou Jing Wai Ke Za Zhi.

[25] Tao P., Amott D., Mitchell P., Iseli T.A. (2017). Massive Epistaxis from Sphenopalatine Pseudoaneurysm 5 Months after Facial Trauma. ANZ J. Surg..

[26] Neres B., Figueiredo E., Aires C., Nogueira E., Andrade E. (2018). Pseudoaneurysm in Internal Maxillary Artery after Gunshot Wound: Critical Review and Case Report. J. Clin. Exp. Dent..

[27] Kim N.Y., Kim J.Y., Pyen J.S., Whan K., Cho S.M., Choi J.W. (2019). Treatment of Pseudoaneurysm of Internal Maxillary Artery Resulting from Needle Injury. Korean J. Neurotrauma.

[28] Al-Saadi N.J., Bakathir A., Al-Mashaikhi A., Al-Hashmi A., Al-Habsi A., Al-Azri F. (2019). Maxillary Artery Pseudoaneurysm as a Complication of Maxillofacial Injuries: Report of Three Cases and Literature Review. Sultan Qaboos Univ. Med. J..

[29] Chaudhary H.A., Ibrahim W.H., Yousaf Z., Abubeker I.Y., Kartha A. (2019). Fungal Malignant Otitis Externa Involves a Cascade of Complications Culminating in Pseudoaneurysm of Internal Maxillary Artery: A Case Report. Am. J. Case Rep..

[30] Chun J.J., Choi C.Y., Wee S.Y., Song W.J., Jeong H.G. (2019). Embolization for Treating Posttraumatic Pseudoaneurysm of the Sphenopalatine Artery. Arch. Craniofac. Surg..

[31] Rawat S.K., Singh D., Suresh Babu P., George R., Mongia P. (2019). Traumatic Pseudoaneurysm: A Life-Threatening Complication After Surgical Extraction of Impacted Maxillary Third Molar. J. Maxillofac. Oral Surg..

[32] Maleux O., da Costa Senior O., Politis C., Maleux G. (2019). Glue Embolisation of a Bleeding Pseudoaneurysm Related to Surgically-Assisted Rapid Palatal Expansion. Br. J. Oral Maxillofac. Surg..

[33] Park B., Jang W.H., Lee B.K. (2019). An Idiopathic Delayed Maxillary Hemorrhage after Orthognathic Surgery with Le Fort I Osteotomy: A Case Report. J. Korean Assoc. Oral. Maxillofac. Surg..

[34] Manoj Kumar K.P., Mullath A., Vijayakumar D., Vinod A. (2021). An Extremely Rare Pseudoaneurysm of Posterior Superior Alveolar Artery Arising after Orthognathic Surgery. Natl. J. Maxillofac. Surg..

[35] Kumar A., Kaur A., Singh M., Rattan V., Rai S. (2021). “Signs and Symptoms Tell All”–Pseudoaneurysm as a Cause of Postoperative Bleeding after Orthognathic Surgery–Report of a Case and a Systematic Review of Literature. J. Maxillofac. Oral Surg..

[36] Słotwińska A., Orzechowska-Wylęgała B., Latusek K., Katra M. (2021). Bilateral Maxillary Pseudoaneurysms as a Complication of Craniofacial Fracture: A Case Report. Am. J. Case Rep..

[37] Hwang J.H., Kim W.H., Choi J.H., Kim K.S., Lee S.Y. (2021). Delayed Rupture of a Posttraumatic Retromaxillary Pseudoaneurysm Causing Massive Bleeding: A Case Report. Arch. Craniofac. Surg..

[38] Miller J.E., McCormick J.P., Raskin J., Borrelli M., Nasrollahi T., Suh J.D. (2022). Endoscopic Management of a Post-Traumatic Internal Maxillary Artery Pseudoaneurysm: Case Report and Review of the Literature. Ear Nose Throat J..

[39] Lim S.Y., Lee H.G., Kim K.N., Kim H., Oh D.H., Koh I.C. (2022). Ruptured Pseudoaneurysm of the Internal Maxillary Artery in Zygomaticomaxillary Fracture: A Case Report. Arch. Craniofac. Surg..

[40] Tokuyama K., Kiyosue H., Shimada R., Miyamoto S., Abe A., Kawano K., Asayama Y. (2023). Selective Transarterial Embolization for Arterial Hemorrhage after Upper Third Molar Extraction: Illustrative Cases. J. Neurosurg. Case Lessons.

[41] Botella-Casas G., Marqués-Mateo M., Miragall-Alba L., Río-Vega D.M., González-Soler E., Puche-Torres M. (2024). Management of Pseudoaneurysms of the Internal Maxillary Artery Derived from Orthognathic Surgery Based on One Case. Oral Maxillofac. Surg..

[42] Lozman H., Nussbaum M. (1982). Aneurysm of the Temporal Artery Superficial. Am. J. Otolaryngol..

[43] Silva A.C., O’Ryan F., Beckley M.L., Young H.Y., Poor D. (2007). Pseudoaneurysm of a Branch of the Maxillary Artery Following Mandibular Sagittal Split Ramus Osteotomy: Case Report and Review of the Literature. J. Oral Maxillofac. Surg..

[44] Lö L., Olmarker A., Geterud K., Risberg B. (2004). Prospective Randomized Study Comparing Ultrasound-Guided Thrombin Injection to Compression in the Treatment of Femoral Pseudoaneurysms. J. Endovasc. Ther..

[45] Cybulak-Naczke J.A., Poleniewicz A., Garbacewicz Ł.M., Polcyn A., Drogoszewska B.T. (2019). Tętniak Rzekomy Tętnicy Szczękowej Jako Rzadkie Powikłanie Złamania Wyrostka Kłykciowego Żuchwy. Mag. Stomatol..

[46] Cox M.W., Whittaker D.R., Martinez C., Fox C.J., Feuerstein I.M., Gillespie D.L. (2007). Traumatic Pseudoaneurysms of the Head and Neck: Early Endovascular Intervention. J. Vasc. Surg..

[47] Radvany M.G., Gailloud P. (2010). Endovascular Management of Neurovascular Arterial Injuries in the Face and Neck. Semin. Interv. Radiol..

[48] Mohanty S., Gulati U., Kathuria S. (2013). Pseudoaneurysm of the Internal Maxillary Artery: A Rare Complication of Condylar Fracture. Craniomaxillofac. Trauma Reconstr..

[49] Rennert R.C., Nguyen V.N., Abedi A., Atai N.A., Carey J.N., Tenser M., Amar A., Mack W.J., Russin J.J. (2023). Combined Open Revascularization and Endovascular Treatment of Complex Intracranial Aneurysms: Case Series. Front. Neurol..

